# Eosinophil Progenitors in Patients With Non-Asthmatic Eosinophilic Bronchitis, Eosinophilic Asthma, and Normal Controls

**DOI:** 10.3389/fimmu.2022.737968

**Published:** 2022-03-31

**Authors:** Chen Zhan, Rong Xu, Bizhou Li, Jiaxing Liu, Wanqin Liang, Shengfang Zhang, Liman Fang, Shuxin Zhong, S. Dushinka Shaniya Helen de Silva, Dhinesan Sivapalan, Wei Luo, Jing Li, Kefang Lai, Nanshan Zhong, Roma Sehmi, Paul M. O’Byrne, Ruchong Chen

**Affiliations:** ^1^ Department of Allergy and Clinical Immunology, State Key Laboratory of Respiratory Disease, National Clinical Research Center for Respiratory Disease, Guangzhou Institute of Respiratory Health, the First Affiliated Hospital of Guangzhou Medical University, Guangzhou, China; ^2^ School of Basic Medical Sciences, Guangzhou Medical University, Guangzhou, China; ^3^ International College of Education, Guangzhou Medical University, Guangzhou, China; ^4^ Department of Medicine, Firestone Institute for Respiratory Health, St. Joseph’s Healthcare and McMaster University, Hamilton, ON, Canada

**Keywords:** non-asthmatic eosinophilic bronchitis, hematopoietic progenitor cells, eosinophil progenitors, airway inflammation, eosinophil

## Abstract

**Objective:**

This study aims to explore the potential of *in situ* airway differentiation of eosinophil progenitors (EoPs) and hematopoietic progenitor cells (HPCs) in sputum and peripheral blood from patients with non-asthmatic eosinophilic bronchitis (NAEB), eosinophilic asthma (EA), and healthy controls (HC).

**Methods:**

Using flow cytometry, we enumerated sputum and blood HPCs and EoPs in patients with NAEB (n=15), EA (n=15), and HC (n=14) at baseline. Patients with NAEB and EA were then treated for 1 month with budesonide (200 μg, bid) or budesonide and formoterol (200/6 μg, bid), respectively. HPCs and EoPs in both compartments were re-evaluated.

**Results:**

At baseline, NAEB and EA both had significantly greater numbers of sputum but not blood HPCs and EoPs (*p*<0.05) compared to HC. There were no differences between NAEB and EA. After 1 month of inhaled corticosteroid (ICS) treatment, NAEB patients showed a significant improvement in cough symptoms, but the attenuation of sputum HPC and EoP levels was not significant.

**Conclusions:**

NAEB patients have increased airway levels of HPCs and EoPs. One-month treatment with ICS did not fully suppress the level of EoPs in NAEB. Controlling *in situ* airway differentiation of EoPs may control airway eosinophilia and provide long-term resolution of symptoms in NAEB.

## Introduction

Non-asthmatic eosinophilic bronchitis (NAEB) is a common cause of chronic cough (1, 2). It is characterized by persistent troublesome cough and airway eosinophilia but lack of airway hyper-responsiveness (3). NAEB patients often respond well to inhaled corticosteroids but frequently relapse (4).

Previous studies have shown that NAEB is a T-helper 2 (Th2)-driven disease. In addition to airway eosinophilia, it was reported that, like asthmatics, NAEB patients have raised levels of inflammatory mediators and cytokines, such as histamine, cysteinyl-leukotrienes, interleukin (IL)-5, and eosinophilic cationic protein (5–7). Persistent sputum eosinophilia is a risk factor for relapse (8), suggesting that airway eosinophilia may be one major pathogenic mechanism in NAEB. Investigating the immunological processes that promote eosinophilic inflammation in the airways is important for the development of novel NAEB therapies.

Mature eosinophils differentiate from eosinophil-lineage committed progenitor cells (EoPs), which arise from bone-marrow-derived CD34+ hematopoietic progenitor cells (HPCs) (9). The differentiation and maturation of EoPs was originally thought to be restricted to the bone marrow (10, 11). However, increased numbers of HPCs and EoPs have been detected in the peripheral blood and tissue compartments from atopic subjects (12–15). It has now been proposed that *in situ* differentiation of EoPs can rapidly increase mucosal numbers of mature eosinophils during an inflammatory response, an element that may drive airway eosinophilia (16, 17). This suggests an important role of *in situ* eosinophil differentiation in the pathology of allergic diseases including asthma and allergic rhinitis.

We hypothesized that *in situ* differentiation of EoPs play a potential role in the pathogenesis of airway eosinophilia in NAEB. In the current study, we enumerated levels of HPCs and EoPs in induced sputum and peripheral blood from NAEB patients compared to eosinophilic asthmatics (EAs) and normal healthy controls (HCs). In addition, we repeated these measurements in NAEB and EA patients after 1 month of treatment with inhaled corticosteroids (ICS) or ICS plus long-acting beta-agonists (LABA), respectively.

## Materials and Methods

### Study Design and Participants

Fifteen patients with NAEB were recruited in the First Affiliated Hospital of Guangzhou Medical University between June 2016 and May 2017. Fifteen patients with EA and 14 HCs were included as disease and health controls. Pulmonary function, fractional exhaled nitric oxide, sputum differential counts, complete blood counts (CBC), serum IgE, symptom scores, and questionnaires including Asthma Control Test (ACT), Leicester Cough Questionnaires (LCQ), and Visual Analogue Scale (VAS) were recorded. Levels of sputum and blood HPCs and EoPs were enumerated by flow cytometry in all participants. NAEB patients were prescribed budesonide (200 μg, bid), and EA patients were prescribed budesonide/formoterol (200/6 μg, bid) for 1 month. Following this, FeNO, sputum differential count, CBC, and flow cytometric assessments of progenitor cells were re-evaluated in both patient groups.

NAEB was diagnosed according to the Chinese cough guidelines (18). The subjects had (1) persistent cough for more than 8 weeks; (2) a normal chest radiograph; (3) sputum eosinophilia (sputum Eos% ≥ 2.5%); and (4) normal spirometry and normal methacholine airway responsiveness. The subjects with EA were diagnosed according to the GINA 2015 criteria (19). All patients with EA had characteristic symptoms (such as wheezing, shortness of breath, chest tightness, or cough); increased sputum eosinophilia (sputum Eos% ≥ 3%), and >12% forced expiratory volume in 1 s (FEV1) reversibility after short-acting bronchodilator or a positive methacholine provocative test. All patients with NAEB or EA were not currently on any steroid therapy. The healthy controls (n=14) had normal spirometry, negative methacholine provocative test, and no history of respiratory disease, allergies, or systemic disease. All subjects were non-smokers. Subjects were excluded if they had experienced a respiratory infection in the past 4 weeks or had a history of bronchiectasis, chronic obstructive pulmonary disease, or other chronic pulmonary diseases.

### Sputum Induction and Cell Isolation

Sputum was induced by inhalation of an aerosol of hypertonic saline as previously described (20). Sputum samples were processed by selecting the mucus plugs, mixing with 4 parts 0.1% dithiothreitol, then filtered through a 48-mm nylon mesh and centrifuged at 3,000 rpm for 10 min at 4°C. The cell pellet was re-suspended in phosphate-buffered saline (PBS). The sputum supernatants were stored at −80°C. The cell smear was prepared and stained with hematoxylin–eosin stain. The differential cell counts of sputum samples were obtained by counting 400 non-squamous cells. The remaining cells were subjected to immunofluorescence staining and enumeration by flow cytometry.

### PBMC Isolation

Peripheral blood mononuclear cells (PBMCs) were isolated from venous blood by Ficoll density gradient centrifugation. In brief, whole blood was diluted 1:1 with PBS and layered onto Ficoll-PAQUE Plus (GE Healthcare, Marlborough, MA, USA), centrifuged at 1,200*g* and 4°C for 25 min with the brake off. The buffy coat was collected and washed twice (500*g* for 5 min) with PBS. Cell count and viability were quantified in a Neubauer’s chamber with 0.4% Trypan Blue Solution.

### Flow Cytometry

Freshly isolated blood-derived mononuclear cells and sputum-extracted cells were immediately incubated with fluorescence-labeled antibodies to define cell subpopulations. Antibodies (BD Biosciences) used for flow cytometry were FITC-CD45, PE-Cy5-CD34, and PE-CD125. Cells were analyzed by FACSVerse analytical flow cytometry (BD Company, San Diego, CA, USA). The percentage of HPCs (FSC^medium^SSC^low^CD45^dull^CD34^+^) and EoPs (FSC^medium^SSC^low^CD45^dull^CD34^+^CD125^+^) were determined using FlowJo software (BD Biosciences). The absolute numbers of HPCs or EoPs were calculated by multiplying percentage of HPCs or EoPs within R2 (singlets with medium FSC and low SSC) in flow cytometry with the absolute number of lymphocytes in blood routine test or sputum cell counts. The gating strategy is shown in **Supplementary Figure S1**.

### Statistical Analysis

Statistical analysis was performed by using SPSS Version23.0 (SPSS Inc., Chicago, IL, USA). Age, body mass index (BMI), FEV1pred, MMEFpred, FEV1/forced vital capacity (FEV1/FVC), and blood eosinophil were presented as mean ± SD and analyzed by one-way ANOVA followed by the Tukey post-test for multiple comparisons. Sputum differential counts, FeNO, serum total IgE, HPCs, and EoPs levels were presented as median (IQR) and analyzed by Kruskal–Wallis test and Dunn’s *post-hoc* analyses for multiple comparisons. Comparisons of LCQ, VAS, ACT, FeNO, sputum and blood eosinophils, HPCs, and EoPs before and after treatment were calculated using a paired t-test or Wilcoxon matched-pairs sign rank test. Correlations among clinical parameters were computed using a Spearman test. Differences were considered to be statistically significant when *p*<0.05.

## Results

### Demographics and Clinical Characteristics at Baseline

Baseline characteristics of participants in the study are shown in **Table 1**. NAEB and EA patients were significantly older and had a higher BMI than HC controls (all *p*<0.01). Similar to EA, NAEB showed higher levels of FeNO, sputum eosinophils, and blood eosinophils compared with HC (*p*<0.05). No differences of FeNO, sputum, and blood eosinophils were found between NAEB and EA.

**Table 1:** Demographics and clinical characteristics at baseline.

	HC	NAEB	EA
	n=14	n=15	n=15
Sex, F/M	8/6	5/10	9/6
Age, y	29.2 ± 6.4	46.7 ± 14.6#	44.5 ± 10.9#
BMI	20.1 ± 2.5	23.5 ± 2.9*	23.9 ± 3.9*
FeNO. ppb	16.0 (9.0, 23.0)	34.0 (23.0, 61.0) #	50.0 (36.0, 79.0) #
FEV1, pred%	95.6 ± 8.2	100.8 ± 12.8‡	81.6 ± 22.3
FEV1/FVC, %	87.7 ± 8.0	83.4 ± 6.6‡	69.8 ± 12.1#
MMEF, pred%	84.0 ± 24.6	88.7 ± 25.5‡	44.0 ± 21.9#
**Induced Sputum**			
TCC, x10^6^/mL	2.3 (0.8-5.8)	2.8 (1.4-13.2)	2.3 (0.9-11.2)
Neutrophils, %	60.2 (40.5, 65.5)	59.0 (26.0, 77.5)	49.0 (21.5, 78.0)
Macrophage, %	37.5 (23.8, 58.5)	21.0 (8.5, 47.0)	11.8 (1.5, 53.5)
Eosinophils, %	0.2 (0.0, 1.0)	4.5 (3.5, 18.5)#	16.0 (8.2, 37.8)#
Lymphocyte, %	1.5 (0.8, 2.2)	1.5 (1.0, 11.5)	2.0 (1.0, 2.5)
**Blood**			
Eosinophils, %	2.2 ± 1.6	5.2 ± 3.2*	6.4 ± 3.2#
Eosinophils, (10^9^/L)	0.1 ± 0.1	0.3 ± 0.2*	0.4 ± 0.2#
Total IgE (KU/L)	55.7 (27.4, 89.9)	98.7 (50.6, 180.0)	107.2 (67.2, 327.0)

Age, BMI, FeNO, FEV1pred, FEV1/FVC, MMEFpred and blood eosinophil are presented as mean ± SD and analyzed by one-way ANOVA. Sputum different counts and Total IgE are presented as median (IQR); sputum TCC is presented as median (min–max) and calculated by Kruskal–Wallis test. The distribution of sex was calculated by chi-square test. Compared with EA: ‡p < 0.01; compared with healthy control: *p < 0.05, ^#^p < 0.01.

### Airway HPCs and EoPs Are Increased in Patients With NAEB

PBMCs were successful obtained from all subjects for flow cytometry detection. Sputum samples for flow cytometry were obtained from 14 patients with NAEB, 11 patients with EA, and 9 subjects with HC.

**Figure 1:**
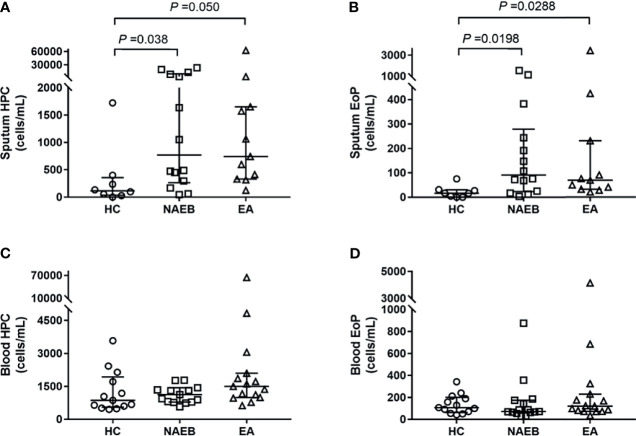
Absolute numbers of hematopoietic progenitor cells (HPC) and eosinophilic progenitor cells (EoPs) in three groups at baseline. Absolute numbers of sputum HPC cells **(A)** and EoP cells **(B)**, and the peripheral blood HPC cells **(C)** and EoP cells **(D)**. Absolute cell numbers enumerated by flow cytometry are presented as cells/ml, and data are presented as median (IQR) values. HC, healthy control group; NAEB, non-asthmatic eosinophilic bronchitis group; EA, eosinophilic asthmatics group.

In the airway, sputum HPCs levels in NAEB [770 (8,219) cells/ml] and EA [742 (1,322) cells/ml] were significantly higher than in the HC group [135 (343) cells/ml] (both *p*<0.05, **Figure 1A**). In addition, sputum EoPs levels were significantly higher in patients with NAEB [91 (219) cells/ml] and EA [69 (199) cells/ml] compared to the HC group [17 (26) cells/ml] (both *p*<0.05, **Figure 1B**). In contrast, there were no significant differences in sputum HPCs or EoPs levels between NAEB and EA. Overall, there was a strong correlation between sputum HPCs and EoPs levels (r=0.778, *p*<0.001, **Supplementary Figure S2**) and a moderate association between sputum EoPs and eosinophils levels (r=0.378, *p*<0.05, **Supplementary Figure S2**).

In the blood, there was no significant difference in HPCs or EoPs levels in NAEB [HPCs, 1,123 (650)/ml; EoPs, 71 (110)/ml] compared with the HC [HPCs, 952 (1,246)/ml; EoPs, 118 (117)/ml] or EA group [HPCs, 1,498 (1,109)/ml; EoPs, 121 (156)] (**Figures 1C, D** **)**. Neither blood HPCs levels nor EoPs levels correlated with blood eosinophil, sputum HPCs, or EoPs levels (**Supplementary Figure S1**).

### HPCs and EoPs Levels Following Treatment With ICS for NAEB or ICS/LABA for EA

**Figure 2:**
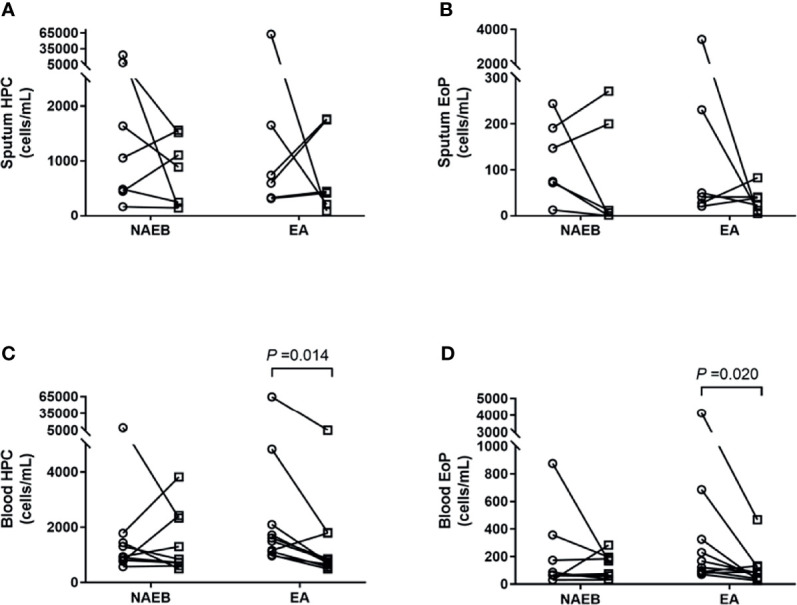
Enumeration of hematopoietic progenitor cells (HPC) and eosinophilic progenitor cells (EoPs) in sputum and peripheral blood after 1 month of ICS treatment in NAEB and ICS/LABA treatment in EA. The changes in sputum HPC counts **(A)** and EoP counts **(B)**, and blood HPC counts **(C)** and EoP counts **(D)** before (open circle) and after (open square) therapy. NAEB, Non-asthmatic eosinophilic bronchitis group. EA, eosinophilic asthmatics group.

After 1-month ICS treatment, patients with NAEB presented an improvement in cough symptoms as reflected by a significant improvement of LCQ [16.2 ± 2.4 vs. 20.4 ± 3.5] and VAS [50 (30) vs. 10 (5)] (all *p*<0.05). One patient reported complete resolution of symptoms. In addition, the NAEB group displayed decreased levels of FeNO [59 (53) vs. 25 (11)] and blood eosinophils [0.3 (0.2) vs. 0.2 (0.1)] (all *p*<0.05). There was reduction in the level of sputum HPCs, EoPs, and eosinophils [HPCs, 1,345(11,476) vs. 887(1,370); EoPs, 169(275) vs. 7(200), cells/ml; Eos, 5.0(37.8) vs. 3.8(10.3), %], although this change did not achieve significance (**Figure 2** and **Table 2**).

**Table 2:** Changes of biomarkers after one-month treatment in NAEB and EA Group.

	NAEB (n=9)			EA (n=10)		
	Baseline	Follow-up	*p*-value	Baseline	Follow-up	*p*-value
FeNO, ppb	59(53)	25(11)*	0.020	46.5(57.3)	28.5(51.7)	0.416
LCQ	16.2 ± 2.4	20.4 ± 3.5*	0.049	NA	NA	NA
VAS	50(30)	10(5)#	<0.001	NA	NA	NA
ACT	NA	NA	NA	19.4 ± 3.7	23.3 ± 1.7*	0.014
Sputum Eos, %	5.0(37.8)	3.8(10.3)	0.148	16.4(36)	5(41.6)	0.734
Sputum Eos (10^9^/L)	0.20(0.80)	0.18(0.40)	0.461	0.41(1.70)	0.10(0.70)	0.160
Blood Eos (10^9^/L)	0.3(0.2)	0.2(0.1)*	0.050	0.4(0.2)	0.3(0.2)	0.456
Blood HPC (cells/ml)	939(822)	843(1,726)	0.820	1553(1,697)	775(1,160)*	0.014
Blood EoP (cells/ml)	68(229)	76(143)	0.637	145(328)	84 (90)*	0.020
	**NAEB (n=7)**			**EA (n=6)**		
Sputum HPC (cells/ml)	1345(11476)	887(1370)	0.297	742(2051)	432(1574)	0.900
Sputum EoP (cells/ml)	169(275)	7(200)	0.297	69(294)	31(41)	0.438

FeNO, LCQ, VAS, and ACT were presented as mean ± SD and analyzed by pair t-test. Other variables were presented as median (IQR) calculated by Wilcoxon matched-pairs sign rank test. Absolute cell numbers enumerated by means of flow cytometry are presented as cells per milliliter. Compared with baseline: *p < 0.05, ^#^p < 0.01

In patients with EA, an improvement in symptoms was observed as reflected by an increasing ACT score (19.4 ± 3.7 vs. 23.3 ± 1.7) (*p*<0.05). A significant reduction was found in blood HPCs and EoPs levels [HPCs, 1,553(1,697) vs. 775(1,160); EoPs, 145(328) vs. 84 (90), cells/ml] (all *p*<0.05) but not mature eosinophils. In contrast, no differences were found in FeNO levels or sputum eosinophils, HPCs, or EoPs after treatment in these patients (**Figure 2** and **Table 2**). For the follow-up time point, when data from EA and NAEB patients were combined, the sputum EoPs level did not correlate with sputum HPCs nor eosinophils levels (**Supplementary Figure S2B**).

## Discussion

The current study has demonstrated, for the first time, that NAEB patients have significantly increased levels of EoPs and HPC in the airways when compared to healthy controls. One-month ICS therapy improved symptoms in NAEB patients; however, the reduction in inflammatory indices including airway EoPs, HPCs, and mature eosinophils was not significant. The results suggest that *in situ* airway differentiation of EoPs may be one of the possible pathways mediating the airway eosinophilia in NAEB. In addition, longer treatment strategies may be required to investigate whether normalization of lung inflammatory cells may further improve symptoms of NAEB and chances of future relapse.

Immune cells that contribute to airway eosinophilia in NAEB are not clearly defined. Brightling et al. (6) previously reported that the proportion of BALF IL-4+CD4+ T cells and the number of IL-4+ and IL-5+ cells in bronchial submucosa are significantly higher in NAEB compared to HC controls, suggesting that the Th2 cell may drive airway eosinophilia in these patients. In addition to the traffic of mature eosinophils from the periphery, *in situ* eosinophilopoiesis has been proposed as an additional process that may drive airway eosinophilia in allergic asthma and rhinitis. It can rapidly increase mucosal levels of mature eosinophils during an inflammatory response. Studies have shown that the increase in numbers of sputum EoPs precedes the development of airway eosinophilia after allergen inhalational challenge in asthmatics (21). Cameron et al. (16) found that local IL-5-dependent differentiation of EoPs was observed when nasal biopsies were cultured *ex vivo* with IL-5 or ragweed allergen resulting in a reduction in CD34 immunopositive/IL-5Rα mRNA+ cells and a concurrent increase in the number of MBP immunoreactive cells, likely mature eosinophils. Our data showed increased levels of sputum HPCs, EoPs in NAEB, and a moderate association between sputum EoPs and sputum eosinophilia indicating, that *in situ* differentiation may mediate local increases in mature eosinophil levels in NAEB. Consistent with this hypothesis, NAEB patients had increased concentrations of IL-5 and granulocyte-macrophage colony-stimulating factor (GM-CSF), which could induce HPCs and EoPs differentiation into eosinophils within the airways (6, 22, 23). In addition, recent studies have suggested that HPCs and EoPs may act as pro-inflammatory effector cells following activation by epithelial cell-derived alarmin cytokines (24). Upon stimulation with thymic stomal lymphopoietin (TSLP) and/or interleukin-33 (IL-33), HPCs shown to express TSLPR and ST2 (25) release high levels of IL-5, IL-13, and GM-CSF (26). Whether NAEB has higher levels of alarmin cytokines in the airways and whether sputum HPC and EoP express greater amounts of type 2 cytokines compared to healthy controls remains to be investigated.

In line with previous studies (17, 21), we found that asthmatic patients showed elevated levels of airway HPCs and EoPs. However, no differences in blood EoPs were observed. This might be related to the severity of the asthmatic patients enrolled, as blood and sputum EoPs in severe asthma are significantly higher than in mild asthma (17). In the current study, the asthmatic patients were mild and steroid naive. Our previous data (17) found a 10-fold greater number of sputum EoPs in prednisone-dependent severe asthmatics compared to mild asthmatics, while these cells were comparable in the PB of both subject groups, suggesting that an enhanced eosinophilopoietic environment exits in the airways of severe asthmatics with persistent eosinophilia.

In contrast to the EA group, neither blood HPCs nor blood EoPs in the NAEB group were different when compared to HC controls. We speculated that there may be systemic component involving mobilization of progenitor cells from bone marrow in EA compared to NAEB where the inflammatory responses appear to be localized to the airways. Eotaxin/CCR3 and SDF-1/CXCR4 axes are reported to play a role promoting progenitors release from the bone marrow in EA and might be less prominent or involved in progenitor cell trafficking to the airways in NAEB (27, 28). This is supported by our previous findings where we found no difference in serum levels of eotaxin or IL-5 in NAEB compared to HC, suggesting that NAEB displayed only mild systemic inflammation (21).

After 1 month of treatment with ICS in NAEB, or ICS/LABA in EA, we found an inconsistent relationship between the improvement of symptoms and relief of inflammation. Despite a significant reduction in blood eosinophils, sputum levels of HPCs, EoPs, and mature eosinophil only showed a trend of not a significant decline in NAEB. In addition, a moderate correlation was found between sputum EoPs and eosinophils in these patients; this might fit out with the hypothesis that that local airway levels of EoP may contribute to the airway eosinophilia. Despite that the attenuation of sputum HPC and EoP levels in EA patients after ICS treatment was not significant, ICS treatment may still have a suppression role on local airway eosinophil differentiation to mature cells. The reasons are as follows: first, the weaker correlation between sputum EOS and HPC or EoP might provide indirect evidence that ICS treatment suppresses differentiation of EoPs into mature eosinophils (**Supplementary Figure S2B**). Second, Kim et al. reported that an increase in CD34+ mononuclear cells occurs in steroid-treated nasal polyps (29). A possible mechanism that they proposed is that inhibition of the differentiation of mature cells from progenitors may cause more residing CD34+ progenitor cells in the local tissue. Since we have previously reported a higher rate of recurring episodes after 1 month of ICS treatment compared to 3 months treatment, during a 1-year follow-up in NAEB (30), our current data suggest that a longer treatment strategy may be required to fully investigate whether reduction in local eosinophilopoietic processes may reduce airway eosinophilia and further improve the management of NAEB.

Some limitations of the study should be noted. First, we measured the total numbers of HPCs and EoPs, and the numbers of activated or cytokine-producing HPCs and EoPs were not determined. Second, our current study is an initial observational, small group study that is hypothesis generating, which provides indirect evidence of the existence of *in situ* airway differentiation in NAEB as reflected by the increased sputum EoPs in NAEB and its moderate correlation with sputum eosinophils. The significance of *in situ* airway differentiation of EoPs leading to airway eosinophilia in NAEB needs to be further investigated.

In summary, this study demonstrated that increases in HPCs and EoPs in NAEB are predominantly found in the airways and that these cells may contribute to the airway eosinophilia. One-month ICS treatment improved the symptoms in NAEB but did not fully suppress the airway inflammatory responses.

## Data Availability Statement

The datasets generated for this study can be obtained from the corresponding author upon reasonable request. Requests to access the datasets should be directed to chen_rch@163.com.

## Ethics Statement

The studies involving human participants were reviewed and approved by the Ethic Committee of the First Affiliated Hospital of Guangzhou Medical University. The IRB number of the ethic approval is 2016-52. The patients/participants provided their written informed consent to participate in this study.

## Author Contributions

RS, PB, and RC planned the study and designed the experiments. JXL, WQL, SZ, LF, SXZ, DSHS, DS, and WL cared for the patients and provided clinical information. CZ, RX, and BL performed the experiments. CZ, RX, and BL performed the statistical analysis and wrote the manuscript. JL, KL, NZ, RS, PB, and RC contributed to made critical revision of the manuscript. RS, PB, and RC supervised the work. All authors contributed to the article and approved the submitted version.

## Funding

This study was supported by grants from the National Natural Science Foundation of China (81870079), State Key Laboratory of Respiratory Disease (SKLRD-QN-201702), Guangzhou Science and Technology Project/Nanshan Medical Foundation (202102010349), Incubation Project for Innovation Team of GMU (Grant 2017-159), and Incubation Program of National Science Found for Distinguished Young Scholars (GMU2020-207).

## Conflict of Interest

RS reports grants and personal fees from Genentech Inc, grants and personal fees from Astra Zeneca, grants and personal fees from Teva Pharmaceuticals, personal fees from GSK, all outside the scope of this work; PO’B has grants in aid and speaker’s fees from AstraZeneca, grants in aid from Bayer, Genentech, Merck, and Novartis, and has received speaker’s fees from GSK, Chiesi and Covis, all outside the scope of this work. RC reports grants and speaker’s fees from AstraZeneca GSK, Sanofi and Novartis, all outside the scope of this work.

The remaining authors declare that the research was conducted in the absence of any commercial or financial relationships that could be construed as a potential conflict of interest.

## Publisher’s Note

All claims expressed in this article are solely those of the authors and do not necessarily represent those of their affiliated organizations, or those of the publisher, the editors and the reviewers. Any product that may be evaluated in this article, or claim that may be made by its manufacturer, is not guaranteed or endorsed by the publisher.
