# Supplementary material for: Eosinophil Progenitors in Patients With Non-Asthmatic Eosinophilic Bronchitis, Eosinophilic Asthma, and Normal Controls
Source: Front Immunol. 2022 Mar 31;13:737968. doi: 10.3389/fimmu.2022.737968 (PMC9009386; doi:10.3389/fimmu.2022.737968)
Supplement: Supplementary file 1 [file DataSheet_1.docx]

**SUPPLEMANTARY MATERIAL
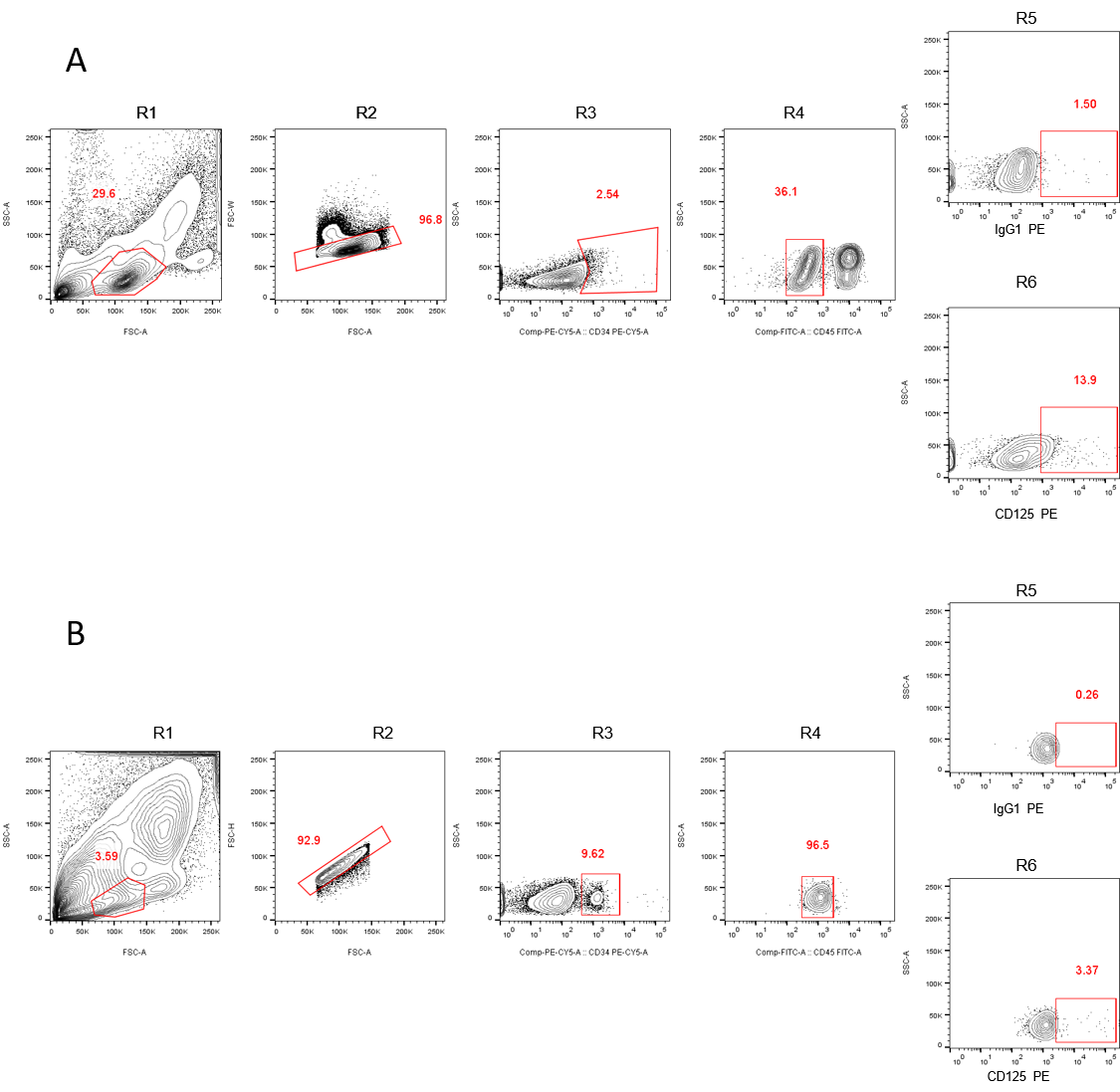
**

**S-Figure 1|** Gating strategy for sputum HPC and EoP enumeration. Representative plots show sequential gating of PBMC(A) and sputum cells(B) to identify region R1(medium FSC and low SSC) and singlets (region R2), HPC was identified as CD45 dull (R4) from the CD34+Cells (region R3), EoP (CD125+) (region R6) was gated from the CD34+CD45dull region and rectangular gate (region R6) was set based on a 98% confidence limit determined by the isotype control (region R5).

**A**


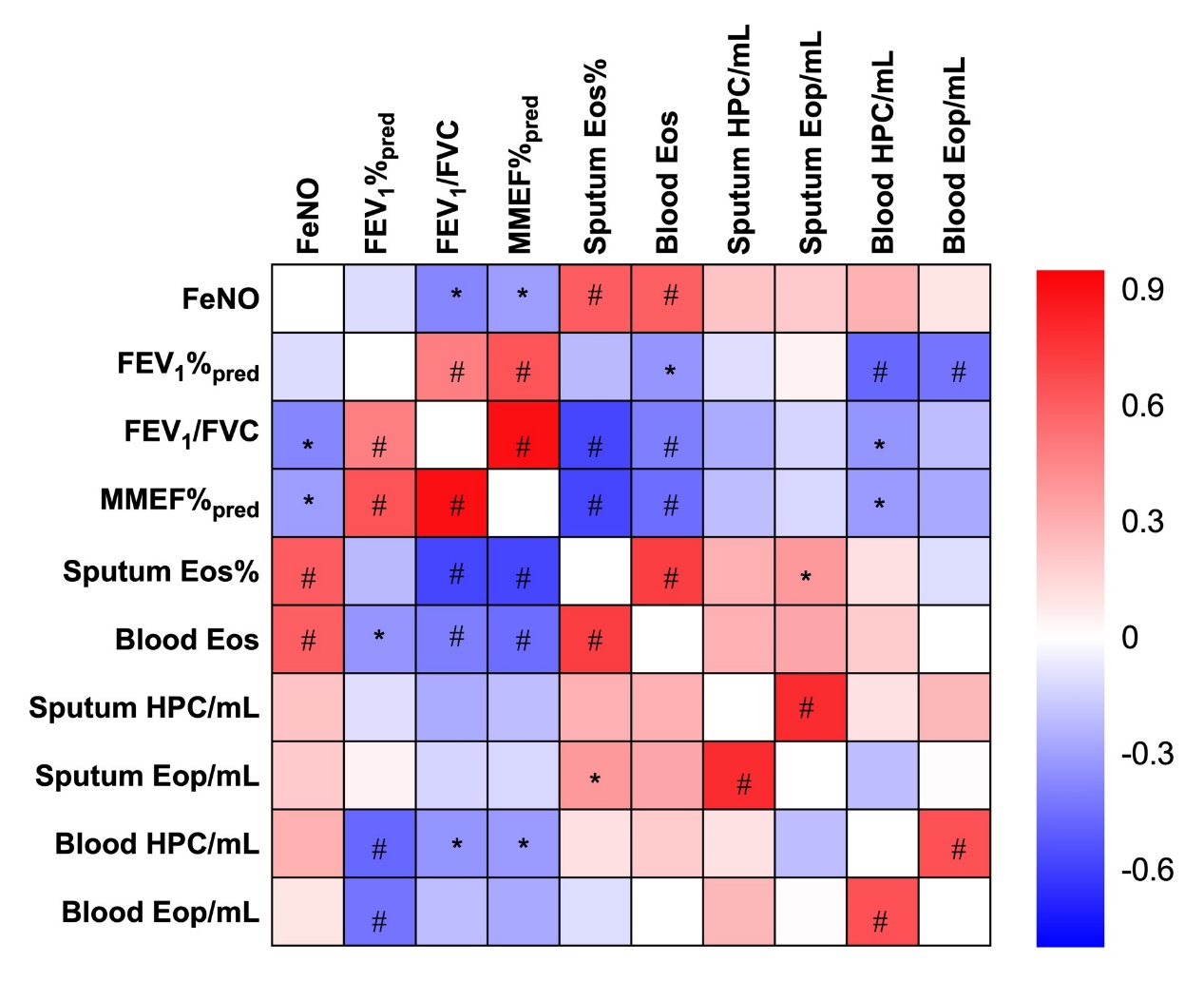


**B**

**
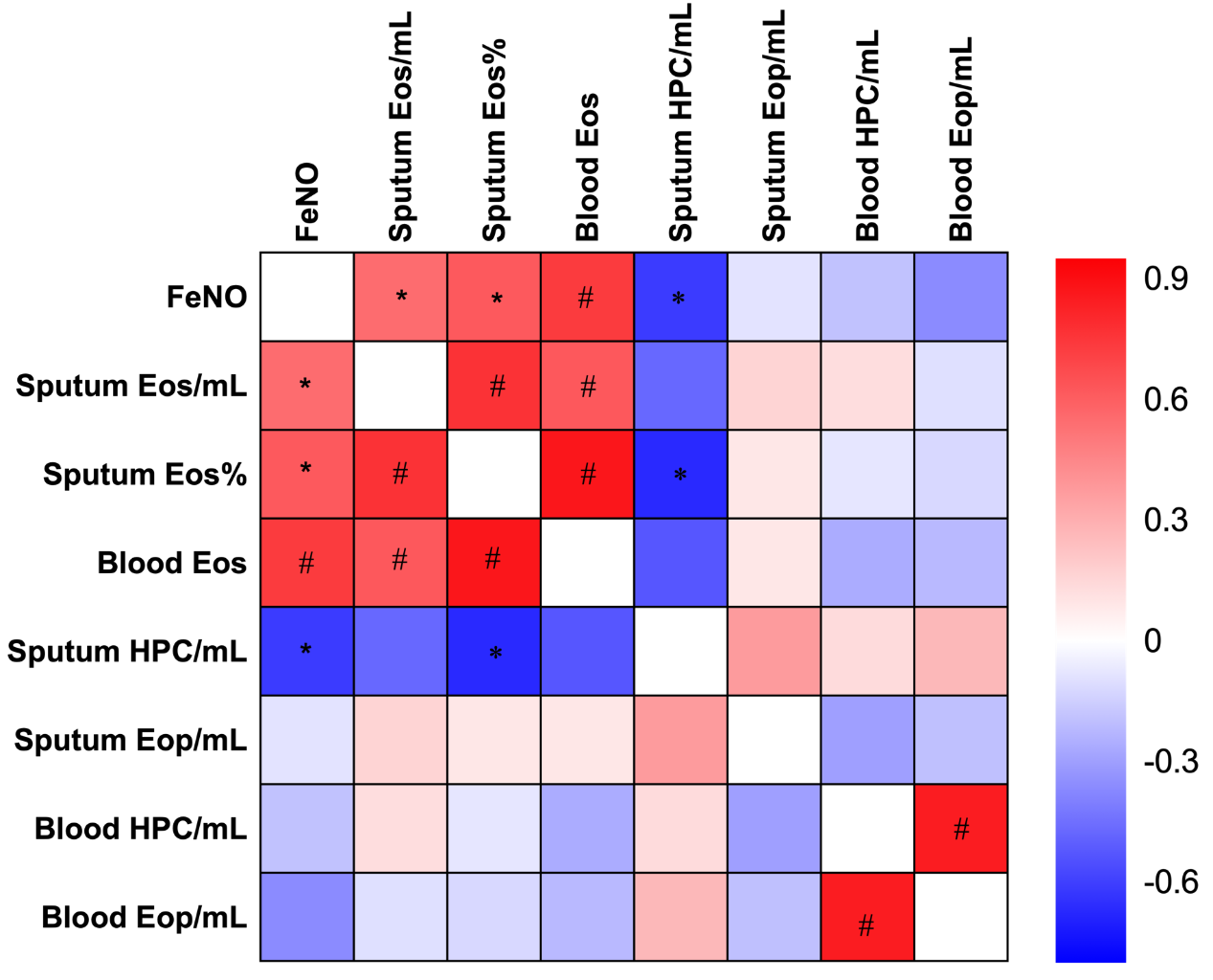
**

**S-Figure 2|** The correlation matrix of laboratory indexes before (A, in total) and after (B, NAEB+EA) one-month ICS treatment. Correlations were computed by spearmen test. The correlation coefficient is represented by color, with blue and red indicating negative and positive relationships, respectively. More correlations were found in the airway inflammatory biomarkers. *: *p*<0.05, #: *p*<0.01.

**S-Table 1-Baseline HPC, EoP cells Level Among Three Groups**

| **Sputum** | **HC(n=9)** | **NAEB(n=14)** | **EA(n=11)** |
| --- | --- | --- | --- |
| Hpcs, cells/mL | 135 (343) | 770 (8219) ^*^ | 742 (1322) ^*^ |
| Eops, cells/mL | 17 (26) | 91 (219) ^*^ | 69 (199) ^*^ |
| Eops/WBC, % | 5.29×10^-4^(1.12×10^-3^) | 3.02×10^-3^(7.46×10^-3^) ^*^ | 1.90×10^-3^(8.91×10^-3^) |
| **Blood** | **HC(n=14)** | **NAEB(n=15)** | **EA(n=15)** |
| Hpcs, cells/mL | 952 (1246) | 1123 (650) | 1498 (1109) |
| Eops, cells/mL | 118 (117) | 71 (110) | 121 (156) |
| Eops/WBC, % | 2.20×10^-3^(1.75×10^-3^) | 1.37×10^-3^(1.29×10^-3^) | 1.82×10^-3^(4.12×10^-3^) |

Data was presented as Median (IQR) and calculated by Kruskal-Wallis test. Absolute cell numbers enumerated by means of flow cytometry are presented as cells per gram/milliliter. Compared with Healthy control: *: p<0.01, #: p<0.01.
